# Growth mindset, delayed gratification, and learning outcome: evidence from a field survey of least-advantaged private schools in Depok-Indonesia

**DOI:** 10.1016/j.heliyon.2021.e06681

**Published:** 2021-04-24

**Authors:** Fandy Rahardi, Teguh Dartanto

**Affiliations:** a Institute for Economic and Social Research (LPEM), Faculty of Economics and Business, Universitas Indonesia, Campus UI Salemba, Jakarta, 10430, Indonesia; b Research Cluster on Poverty, Social Protection and Development Economics, Department of Economics, Faculty of Economics and Business, Universitas Indonesia, Campus UI Depok, Depok, 16424, Indonesia

**Keywords:** Learning outcome, Growth mindset, Delayed gratification, Education policy, Indonesia

## Abstract

Indonesian students perform poorly compared with students in other countries, despite education taking a significant portion of the national budget. Although poor infrastructure is often blamed for this failure, several reports show that it may not be the sole determinant of learning outcomes. Using the concepts of a growth mindset and delayed gratification as proxies for human behavior, we conducted a field survey of four highly disadvantaged private schools in Depok, West Java, Indonesia to observe how human behaviors affect learning outcomes. We use a self-administered mathematics test to measure learning outcomes, and construct a questionnaire based on Dweck's Implicit Theory of Intelligence to measure students’ growth mindset. Delayed gratification is measured using a Convex Time Budget (CTB) questionnaire. Controlling for various student characteristics, our estimations show that a more pronounced growth mindset is associated with better math scores. Meanwhile, delayed gratification has mixed effects on learning outcomes: it has no significant effect when the relationship is estimated using an Interval Censored Regression (ICR), but has some statistical significance when an Ordinary Least Square (OLS) regression is used. These results imply that a student's beliefs on a growing intelligence can affect their academic achievement, whereas the ability to resist temptation has inconclusive effects on academic achievement. This difference may be caused by several factors such as the developmental stage of students. Interestingly, satisfactory teaching practices do not necessarily lead to better math scores. Our findings, therefore, suggest that future education policy design must consider aspects of human behavior in order to more optimally benefit students.

## 1 Introduction

Indonesia has made great strides in improving universal access to education by constitutionally allocating 20% of the annual state budget towards education. In 2019, that amounted to more than IDR 492 trillion (USD 38.48 billion) in 2019. Yet, the increase in spending has not resulted in better learning outcomes (de Ree et al., 2018). A cross country comparison using the 2015 Programme for International Student Assessment (PISA) scores shows that Indonesian students ranked (on average, in the three subjects of reading, mathematics, and science) 62^nd^ out of 69 countries (OECD, 2016). Indonesia also performs worse than its regional neighbors like Vietnam and Thailand. Moreover, a large discrepancy remains between students’ numerical abilities and what they are supposed to know based on the education curriculum, with students showing no substantial increase in numeracy skills even after several years of schooling (Beatty et al., 2018).

With gross enrollment of primary and secondary education having reached 103% and 86% respectively, Indonesia's biggest challenge regarding education is no longer improving access; rather, the country faces a dire need to improve the quality of education. Azzizah (2015) argues that inequality between regions and the lack of infrastructure can explain the poor learning outcomes of Indonesian students, whereas the OECD/Asian Development Bank (2015) cites other conventional factors such as human resource deficits, perverse incentive structures, and poor management. Generic interventions such as increasing education funding, improving teacher training, and reforming the education administration may raise the quality of education. However, those interventions largely focus only on students’ external conditions, and not specifically on the students themselves who are the main targets in the effort to enhance learning outcomes. In designing and implementing policy reforms, it is critical that policymakers do not overlook the role of student conditions and characteristics as students are the treatment-receiving subjects.

Hence, direct interventions into student behaviors such as addressing the psychological conditions of a growth mindset and delayed gratification may improve learning outcomes. Recent developments in psychology have shown that non-cognitive factors can influence a student's academic achievement. One of these non-cognitive factors involves the implicit theory of intelligence (ITI), which refers to underlying beliefs regarding whether intelligence or ability can be changed (Dweck and Leggett, 1988). Research has shown that both types of ITI – entity beliefs and incremental beliefs – are linked with students’ academic achievements. Entity beliefs refer to the belief that intelligence is fixed, and they are positively associated with verbal and quantitative domains (Pepi et al., 2015). Meanwhile, incremental beliefs are held by those who believe that intelligence and ability are malleable; Costa and Faria (2018) find that individuals who believe in the malleability of intelligence are more likely to have both higher grades in specific subjects and higher overall achievement. This growth mindset towards intelligence has repeatedly been shown to significantly affect the academic achievement of students (Blackwell et al., 2007; Romero et al., 2014).

In addition to the growth mindset, the education literature also discusses the notion of delayed gratification, which is defined as the ability to resist the temptation of an immediate reward for a later reward – generally, the later reward is a larger one. In education, the ability to delay gratification is essential as students might have to resist immediate temptations in favor of longer but more valuable academic goals (e.g. resisting the temptation to play in order to prepare for an exam). Students who are unable to resist immediate temptations may procrastinate less palatable tasks despite long-term academic rewards to completing those tasks. Several studies have shown that procrastination and the inability to delay gratification are correlated with worse academic achievements (De Paola and Scoppa, 2015; Watts et al., 2018). These studies suggest that interventions to shift students towards growth mindsets while simultaneously promoting behaviors of delayed gratification can result in better learning outcomes.

Few interdisciplinary studies address Indonesia's complex problems in education, especially from the human behavior perspective. Thus, this study aims to observe how student behaviors such as growth mindsets and delayed gratification (or ability to resist temptation) can predict variations in a learning outcome among Indonesian students, specifically those in low-performing and highly disadvantaged private schools. We therefore conduct a small field survey in four of the disadvantaged private junior high schools at the eighth-grade level to examine whether there exists significant relationships between growth mindsets, delayed gratification, and learning outcomes in the specific form of mathematical competency.

We intentionally choose these schools referring to the result of national exam. The 2017 national exam results in Indonesia show that a majority of junior high school students scored below passing grade^1^, with students in private schools (54% of their students scoring below passing grade) performing slightly worse than students in public schools (52%). As such, this study deliberately chooses four private schools in Depok-West Java that have relatively similar qualities with most private schools in Indonesia.

Finally, this study not only contributes to interdisciplinary research in education, economics, and psychology, but also complements existing studies to seek an effective and efficient policy to improve upon the quality of education in Indonesia. The results of this study can provide context for future policymaking in education about the role of student behaviors. If this study can prove that a growth mindset and delayed gratification are essential factors in determining student learning outcomes, it can be scaled up and then used as a national policy to enhance existing policies in reforming education.

This study proceeds as follows: Section 1 explains the study's background. Section 2 provides a literature review on the relationship between growth mindsets and delayed gratification, along with their impacts on learning outcomes; Section 3 presents the methodological framework, survey design, data, and econometric estimation procedures used. Section 4 analyses the findings for a relationship between students’ growth mindsets, tendencies regarding delayed gratification, and learning outcomes in terms of mathematical competency. Lastly, the concluding section of the paper summarizes the key findings and discusses policy implications and limitations.

## 2 Literature review

The role of mindsets in education was first introduced by Dweck (2006). A mindset generally refers to an individual's beliefs about how they think of certain issues. Yeager and Dweck (2012) classify the concept of a mindset into two categories: a fixed mindset and a growth mindset. A fixed mindset is the belief that intelligence is somewhat given and efforts to improve intelligence are likely to be futile, while a growth mindset is the belief that intelligence can be changed and improved through rigorous effort. Schroder et al. (2017) show that people with a fixed mindset tend to focus on their mistakes, especially in challenging circumstances. From an education perspective, students with a fixed mindset tend to choose easy problems as they need to "prove" their intelligence, whereas those with a growth mindset are more eager to accept challenges because they believe doing so can improve their intelligence. As people tend to improve their performance when faced with a difficult situation, this belief is important in education because the learning process is the core of all educational activities.

Blackwell et al. (2007) have shown that – compared to those with a fixed mindset – students categorized as having a growth mindset achieve higher math scores, especially after facing challenges and setbacks. In poor societies, students’ growth mindsets can also compensate for their lack of academic achievement (Claro et al., 2016) and students with a growth mindset were reported to experience a slower decline in their test scores (McCutchen et al., 2015). A growth mindset can improve the GPAs of underachieving students (Paunesku et al., 2015) and prevent students from focusing too much on making mistakes, thereby preventing inhibition of the learning process (Schroder et al., 2017). Meanwhile, Burnette et al. (2017) and Dixson et al. (2017) each found that a growth mindset intervention was effective in improving the learning achievements of rural adolescent girls and African-Americans.

However, several studies show that the importance of a growth mindset may be less than research has suggested. For example, the impact of a growth mindset may be small, or negligible, in improving the academic performance of students who are already high-achieving (Orosz et al., 2017). Additionally, while a growth mindset may improve academic achievement in the short run, the effect fades when the spectrum of time is extended or when older students such as university students are the subject of the intervention (Bahník and Vranka, 2017). Hence, the net effect of a growth mindset must be considered carefully, especially when other non-cognitive factors come into play.

Another such non-cognitive, behavioral factor that can affect learning outcomes is delayed gratification. Delayed gratification refers to a person's ability to delay immediate rewards in favor of later and better rewards (Drobetz et al., 2014). The absence of delayed gratification is also known as present-biased preference^2^. People with present-biased preference tend to be more impatient and more willing to make a decision that benefits them instantly, even if that decision yields a significantly lower result in terms of amount. As they put more value on immediate rewards, the inability to delay gratification can often lead to procrastination, especially if doing an action requires immediate costs (O'Donoghue and Rabin, 1999; Reuben et al., 2015). Numerous studies have explored how the absence of delayed gratification, or having present-biased preferences, is associated with lower academic performance (Golsteyn et al., 2014; Non and Tempelaar, 2016). A field experiment conducted by Ariely and Wertenbroch (2002) shows the problem of self-control is associated with lower task performance, even for high-achieving students. These findings suggest that the inability to delay gratification is adversely associated with academic achievement.

## 3 Research method

### 3.1 Design of survey

We designed a field survey to test whether a growth mindset and delayed gratification have significant effects on student learning outcomes by using math test scores as the proxy for the dependent variable of learning outcomes. The survey was conducted in four private junior high schools (Sekolah Menengah Pertama/SMP) in Beji District, Depok, West Java from early March to late April 2018, between 8 a.m. to 10 a.m. The selection criteria was low-performing schools, indicated by national math exam scores lower than the national average^3^ of 50.19 (Ministry of Education and Culture of Indonesia, 2020), and disadvantaged schools with a lack of adequate supporting infrastructure, as indicated by the low percentage of certified teachers in the school^4^ (Ministry of Education and Culture of Indonesia, 2018).^5^ We specifically chose low-performing schools as they should broadly represent the education landscape in Indonesia; the 2015 PISA test indicates that Indonesian student performance is among the lowest in PISA-participating countries (OECD, 2019). The 2017 national exam results further strengthen this argument: more than half (53%) of students in Indonesia scored less than the passing grade of 55 set by the Ministry of Education. We exclude public junior high schools due to the limited number of public schools within the area. This exclusion means the results of this study should be generalized only to low-performing private schools and not to the overall education landscape in Indonesia. Using only math test results to measure learning outcomes also implies that this study should not be generalized for overall learning outcomes, since results may be differ for other subjects such as science and reading.

The respondents selected for this survey were junior high school students in the eighth grade, chosen because seventh grade students were less likely to understand the instructions, while ninth grade students were occupied in their preparation for the national exam. Furthermore, the age profile of eighth-grade students, which will be shown in greater detail in the Descriptive Statistics, is similar with the age profile of students in the PISA test, i.e. 15-years-old students. The study's sample size was calculated using Lehr's (1992) formula:

(1) $$Total\;Sample\;Size=\frac{16}{EffectSize^{2}}$$

The intended effect size was between 0.2 to 0.25, making the total sample size between 256 to 400 students. 343 eighth-grade students from four different schools qualified for this study. To ensure that participants are willing to join the survey and meet the required ethical procedures for under-aged research subjects, we distributed a parental consent form before the start of the survey.^6^

This research was carried out over a three-week period. In the first week, students were given the parental consent form. The students or their parents could retract their consent to participate at any time during the survey for any reason, such as if they believed that the research posed any harm to them. The signed form was given back to the surveyor in the second week and students were given two questionnaires that measured their time preference and mindset characteristics.

### 3.2 Delayed gratification

To measure tendencies of delayed gratification, we adopt a modified Convex Time Budget (CTB) (Andreoni et al., 2015) questionnaire. The CTB measures temporal discounting, i.e. the tendency for people to prefer immediate, albeit smaller, rewards over larger but delayed rewards (Kirby and Marakovic, 1996). We use the CTB instead of other elicitation methods such as multiple price lists (MPL) because our pilot study suggested that the CTB is more easily understood by eighth-grade students. Students faced 24 convex budget decisions, which involved four combinations of starting times, *t*, and delays between payments*, d.* Two earlier payment dates, *t* = (0, 14) and two delays between payments, *d* = (14, 28) are crossed with six different values of interest rates, *r* = (0%, 11%, 25%, 42%, 66%, and 100%). The earlier payment dates and the delays were structured to ensure that payments would be delivered before the end of the ongoing academic term, even for payments with the longest schedules. For each question, students were given a budget, *Y,* of IDR 40,000 or approx. USD 3. Payments received in earlier periods are denoted as $C_{t}$, while those received later are denoted $C_{t+k}$, with $C_{t}\left(1+i\right)$ + $C_{t+k}$ equaling to IDR 40,000. Students must decide, in each question, the budget allocation that they will receive. There were five budget allocation options for student to choose from: (1) 100% of the budget allocated earlier (received at *t* days after the completion of the survey); (2) 75%; (3) 50%; (4) 25%; and (5) none, i.e. 100% of the reward allocated later (received at *t* + *d* days).

To measure the ability to resist temptation, we measured students’ degree of present bias, β. Parameters of present bias are elicited through a time-separable CRRA utility function, via the quasi-hyperbolic discounting function (Laibson, 1997). Assuming students are maximizing their utility subject to an intertemporal budget constraint (Equation 2),

(2) $$\left(1+r\right)c_{t}+c_{t+k}=Y$$

where Y is the endowment, r is the gross interest rate (1 + r = Y/C), and c_t_ is the payoff at period-t. The relationship can be rearranged to be a linear function:

(3) $$ln{\left(\frac{C_{t}-\omega_{1}}{C_{t+k}-\omega_{2}}\right)}_{ij}=\frac{ln\beta }{\alpha -1}.{1_{t=0}}_{ij}+\frac{ln\delta }{\alpha -1}.k_{ij}+\left(\frac{1}{\alpha -1}\right).ln{\left(1+r\right)}_{ij}$$

where α is the parameter of the CRRA utility curvature, $\omega_{i}$ is the additional utility parameter and $1_{t=0}$ is a dummy variable which equals to 1 when t is equal to 0. In this study, $\omega_{i}$ is assumed to be 0. The parameters of α,δ, and β can be determined using the Ordinary Least Squares (OLS) and the Interval-Censored Regression (ICR). However, due to the limited number of budget allocation options, the ICR technique is more appropriate. Results using the OLS technique are also displayed for comparative purposes, but the Non-linear Least Squares (NLS) technique could not be conducted due to the non-convergent utility function. Students were said to be present-biased if β < 1 whereas β > 1 indicates future-biasedness. A higher β reflects delayed gratification or a better ability to resist temptation. The association between delayed gratification and learning outcomes (Horn and Kiss, 2017) is then analyzed through the following model:

(4) $$MathSc_{i}=\alpha_{0}+\alpha_{3}{\left(\hat{\beta }\right)}_{i}+\delta_{ik}Control_{ik}+\varepsilon_{i}$$

where $\hat{\beta }$ is the estimated parameter of present bias. This model aims to determine whether a larger $\hat{\beta }$, which implies future-biasedness or the existence of delayed gratification, is associated with higher learning outcomes. One of the issues that arises in time-preference elicitation is whether participants believe that the payments will be paid in the future. To maintain confidence, we explained that the payments were guaranteed to be delivered, even if participant were absent during the scheduled the payment, the payment would be given to them through their school teacher. They could also notify researchers directly through the contact listed on the consent form if they did not receive the payment when due. All participants received two chocolate bars as compensation for their participation, and two students from each class were randomized to receive their payments based on their choices. The announcement and the reward distribution were done after the mindset measurement.

### 3.3 Growth mindset elicitation

The measurement of students’ mindsets occurred in the last part of the second week. This study used a questionnaire based on the Implicit Theory of Intelligence Scale (ITIS) to measure each student's mindset (Dweck and Leggett, 1988). Research has indicated that ITIS has good reliability (α ranging from 0.82 to 0.97) and construct validity (Dweck et al., 1995), which is also true in the case of gifted students (Park et al., 2016) or when presented in different languages (Omidmehr et al., 2018). Participants answered 20 items using a 4-point Likert scale; from “Strongly disagree” to “Strongly agree”. Each answer, depending on the question, is assigned a value from 0-3. For example, strong agreement with the statement “you can always change your basic intelligence” receives the highest value of 3, while strong agreement with “you cannot change your basic intelligence” receives the lowest value of 0. The final score is the sum of all values from 20 items, with higher scores corresponding to a growth mindset and lower ones reflecting a fixed mindset.

As the original question was written in English, we translated the questions into Bahasa Indonesia because not all students understood English. To ensure that the translation can be understood by eighth-grade students, we test the initial (translated) questionnaire on several eighth-grade students and adjust the translation based on the pilot results before using it for the main survey. To determine whether there was a difference in the proportion of students with a growth mindset across schools, a Chi-square test, Monte Carlo Simulations, and the Marascuillo Procedure were utilized to determine whether at least one school had a statistically different proportion of students with a growth mindset and, if so, which schools stood out.

In the final week, the students were required to fill out a four-page questionnaire^7^. The first page asked questions regarding students’ demographic backgrounds, the second page asked questions related to economic factors or resources. Questions about more personal information such as the subjects they preferred and disliked the most, the year they entered junior high school, and their national math scores in elementary school were put in the third page. Information regarding the subjects they preferred and disliked the most was used to determine their attitude towards math. The last page contained questions related to students’ perspective on learning, such as how well they think they understand mathematics taught at school, how difficult they find their class content, and how important the quality of supporting infrastructure is to them.

In the final part of the study, students were required to answer several questions about mathematics to measure their academic performance. The test consisted of twenty questions in a 30-minute session. The grading was done in a 0–100 scale, and each correct answer was given 5 points. The score obtained from the mathematics test was used as the proxy for educational outcomes. We use a self-administrated test as the difficulty of school-administered exams may vary between schools. Although the test itself was administered near the end of the academic term, our pilot study suggests that the amount of materials taught in each school at a similar time might be slightly different. As such, the problem set was designed with questions based on the national curriculum (K-13) and covers materials from elementary-school up to the first term of eighth-grade, to prevent any biases in scores that could arise from the differences in the amount of materials taught in each school. Choosing a problem set with a lower level of difficulty also better reflects the intent of this study because learning outcomes will be more appropriately measured with topics that students have studied before.

The problem set was comprised of four parts: the first contained short answer questions, the second contained multiple-choice questions, the third contained a case-study-based problem set, and the fourth contained more short answer questions but with a higher level of difficulty (topics for the first semester of eighth-grade). After the mathematics test, students were given a token of appreciation in the form of a notebook and a keychain. To determine whether potential selection bias arose, statistical analyses of students’ math scores were conducted using a One-way ANOVA and a Tukey HSD to determine and identify differences in average math scores across schools.

We developed a simple econometric model to test whether possession of a growth mindset has a significant relationship with learning outcomes. The dependent variable used was students’ math scores, and two types of the explanatory variable were used: the degree of students’ growth mindsets and a dummy variable for the growth mindset (Blackwell et al., 2007; Claro et al., 2016; Paunesku et al., 2015). The first model is shown as follows,

(5) $$MathSc_{i}=\beta_{0}+\beta_{1}.MindSc_{i}+\overset{n}{\underset{k=1}{\sum }}\delta_{ik}Control_{ik}+\varepsilon_{i}$$

where $MathSc_{i}$ and $MindSc_{i}\;$ are the math score and mindset score for individual *i*, and $Control_{ik}$ is the vector of control variables. In constructing a dummy variable to indicate a growth mindset, the cutoff point of 45 was chosen for the mindset score.

The control variables included in this model are respondent age (Melkonian and Areepattamannil, 2017), gender (Melkonian and Ierokipiotis, 1997), size of extended family (Maralani, 2008), amount of electronic-based resources in students’ homes (Vigdor et al., 2014), students’ attitude towards school subjects (Nicolaidou and Philippou, 2003), student satisfaction of teacher pedagogies (Coe et al., 2014), quality of the classroom (Rivkin and Schiman, 2015), and a categorical dummy for the school (Newhouse and Beegle, 2006).

## 4 Results and discussion

### 4.1 Descriptive Statistics

Most students were aged 14 or 15 by the time the data was recorded (see Table 1). The distribution of gender was almost equal; 55% of the students surveyed were male. There are some differences in the number of students surveyed from each school, with *School ID 2* (113 students) contributing the most students and *School ID 1* (60 students) contributing the least. On average, the number of family members is 5.24 persons (SD = 2.77). 89% of students have access to handphones at home, and almost 50% own a computer at home. Approximately 47% of students (SD = 0.49) stated that they hate mathematics. Students on average are satisfied with their teachers, with an average satisfaction score of 3.65 (SD = 0.73) from the scale of 5. Similarly, students feel that the quality of their classes are relatively good, with an average satisfaction score of 3.51 (SD = 0.74) from a scale of 5.

**Table 1:** Demographic background.

Variable	N	%	Variable	n	Mean	SD
**Age**			**Other demographic variables**			
<13	82	26.03	Number of family members	343	5.241	(2.776)
14	157	49.84				
15	64	20.32	Dummy for having a handphone (Yes = 1)	343	0.895	(0.307)
>16	12	3.81				
**Gender**			Dummy for having a computer (Yes = 1)	343	0.478	(0.500)
Male	190	55.39				
Female	153	44.61	Dummy for negative attitude toward mathematics	343	0.472	(0.499)
**School**						
ID 1	60	17.49	Student’s satisfaction on teacher’s pedagogy (scale of 1–5)	326	3.656	(0.730)
ID 2	113	32.94				
ID 3	75	21.87	Student’s perspective on class condition (scale of 1–5)	323	3.507	(0.745)
ID 4	95	27.70				

Source: Authors’ estimation.

**Figure 1:**
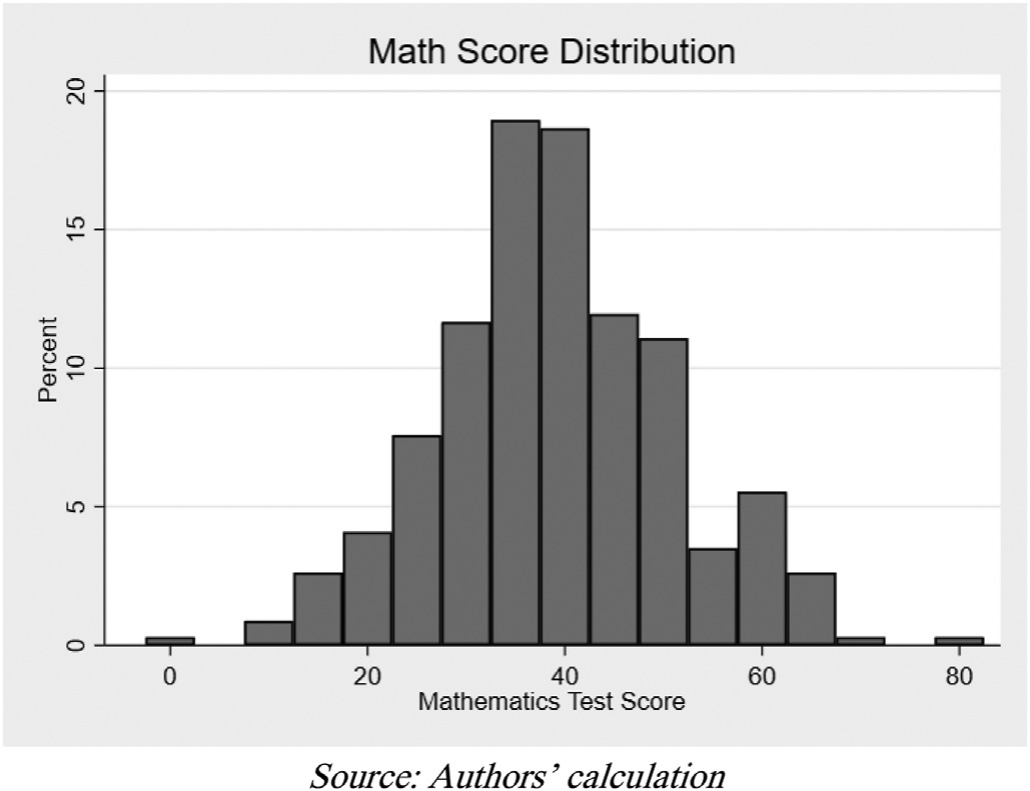
Distribution of mathematics test result.

Of the 343 students who participated in the test, the average math score was 39.05 (Figure 1). Considering that the large portion of the questions were at an elementary school level, the test results are arguably low for junior high school students. This poor result may be because students forget what they learn at the elementary level. However, as almost half of the questions only required basic arithmetic skills (addition, subtraction, multiplication, and division), this result poses a question of whether students understand what they learn during elementary school, and is similar to findings from Beatty et al. (2018), who showed that high school graduates struggled to correctly answer numeracy problems that they should have mastered in elementary school.

**Figure 2:**
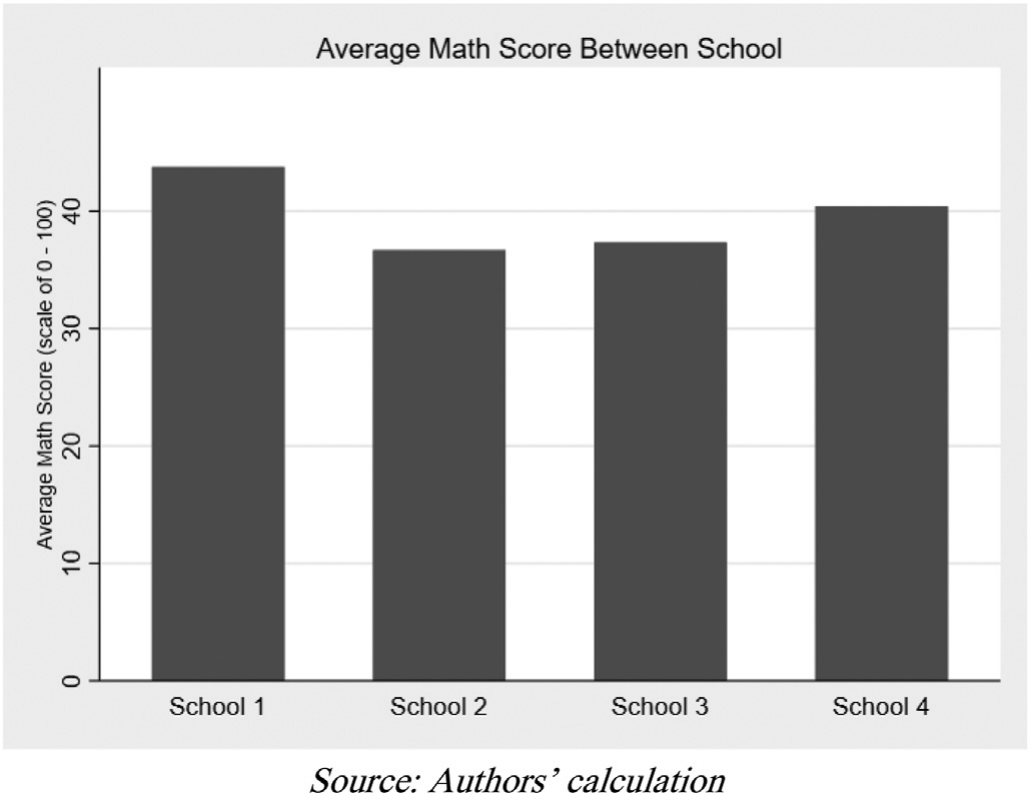
Average math score between schools.

Figure 2 shows the average math score across schools. The ANOVA indicates significant differences in the average math scores between schools, and the Tukey HSD shows that the differences are between *School-1* and *School-2*, and between *School-1* and *School-3*. These differences may arise due to the varying quality of students in each school and imply that controlling for the school is necessary in the regression analysis to eliminate these biases.

**Figure 3:**
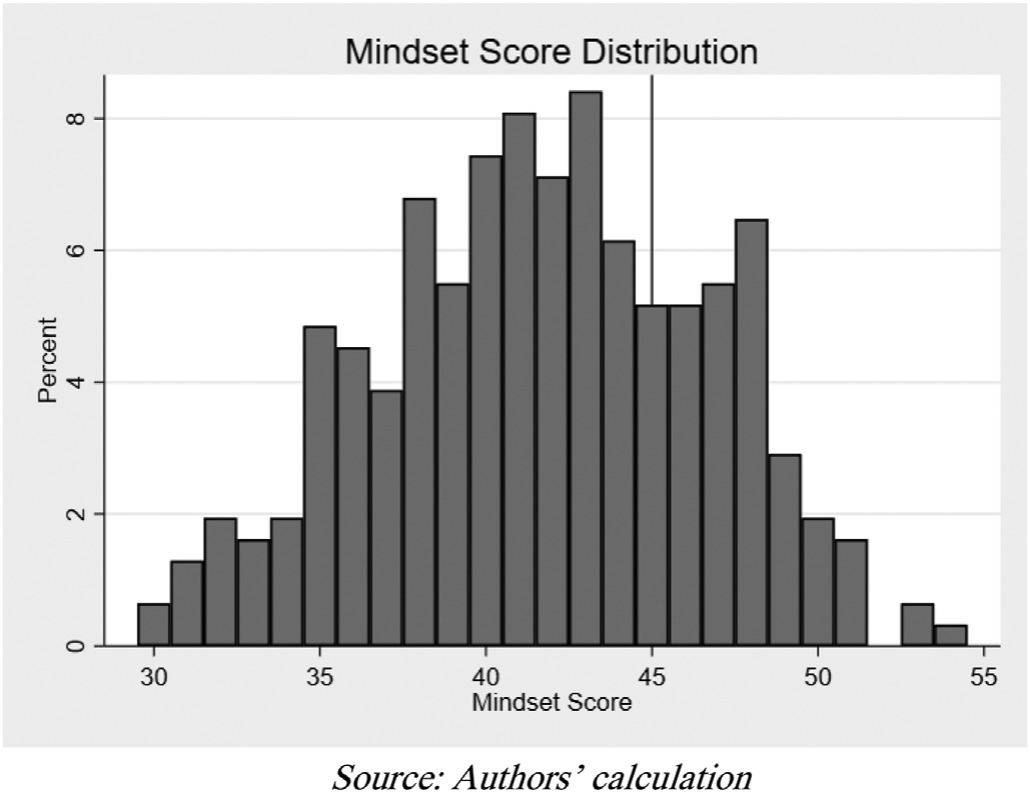
Distribution of mindset score.

**Figure 4:**
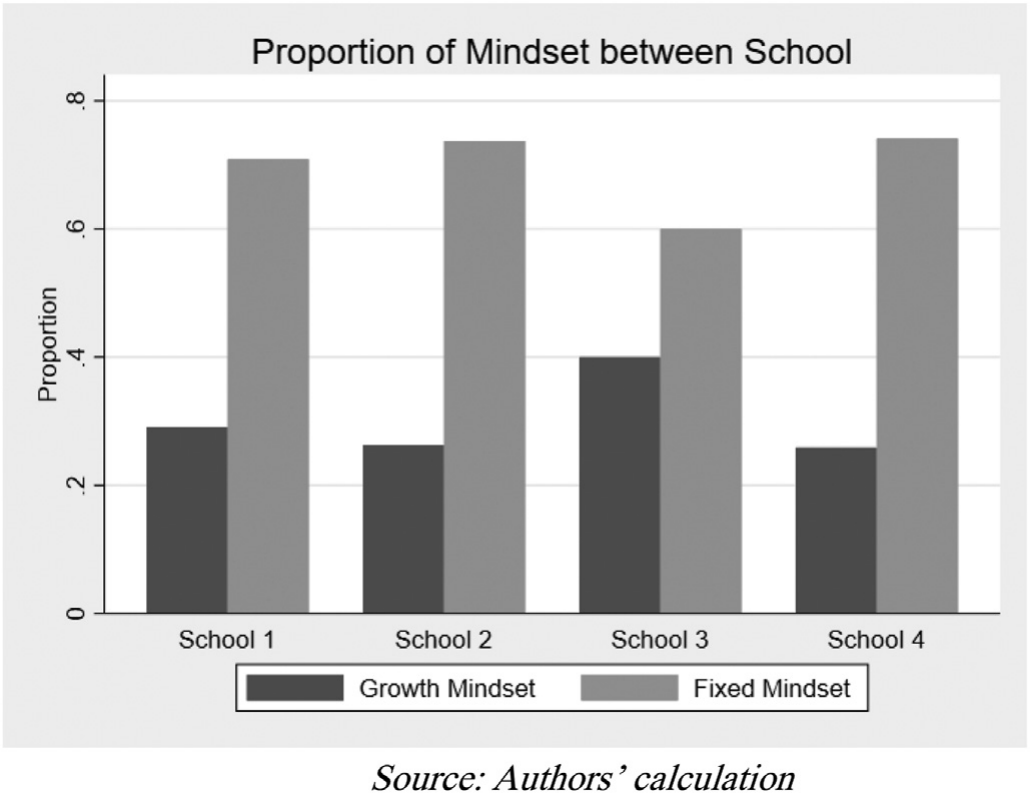
Proportion of student mindset between schools.

Figure 3 presents students’ mindset score distribution. From the scale of 0–60, the average mindset score was 41.66, with the lowest score being 30 and highest being 54. By setting 45 as the cut-off point to indicate a growth mindset, 92 students are considered to have a growth mindset (26.82% of total students). Meanwhile, Figure 4 shows the proportion of students with a growth mindset in each school. Using the Chi-square test, we did not find statistical evidence of differences in the proportion of students with a growth mindset across schools (Pearson $x^{2}$ = 4.71; Prob = 0.194). Other statistical tests such as Monte Carlo simulations and the Marascuillo procedure also found no significant differences.

Students’ ability to delay gratification were measured according to their allocation choices for monetary rewards across different times, *t*. Students are present-biased or unable to resist immediate temptation if they allocate a larger proportion for front-end payments. The consistency of students’ answers was measured by comparing their answers across different discount rates. The calculations find that the choice consistency^8^ at the individual level is 69.38%.

**Figure 5:**
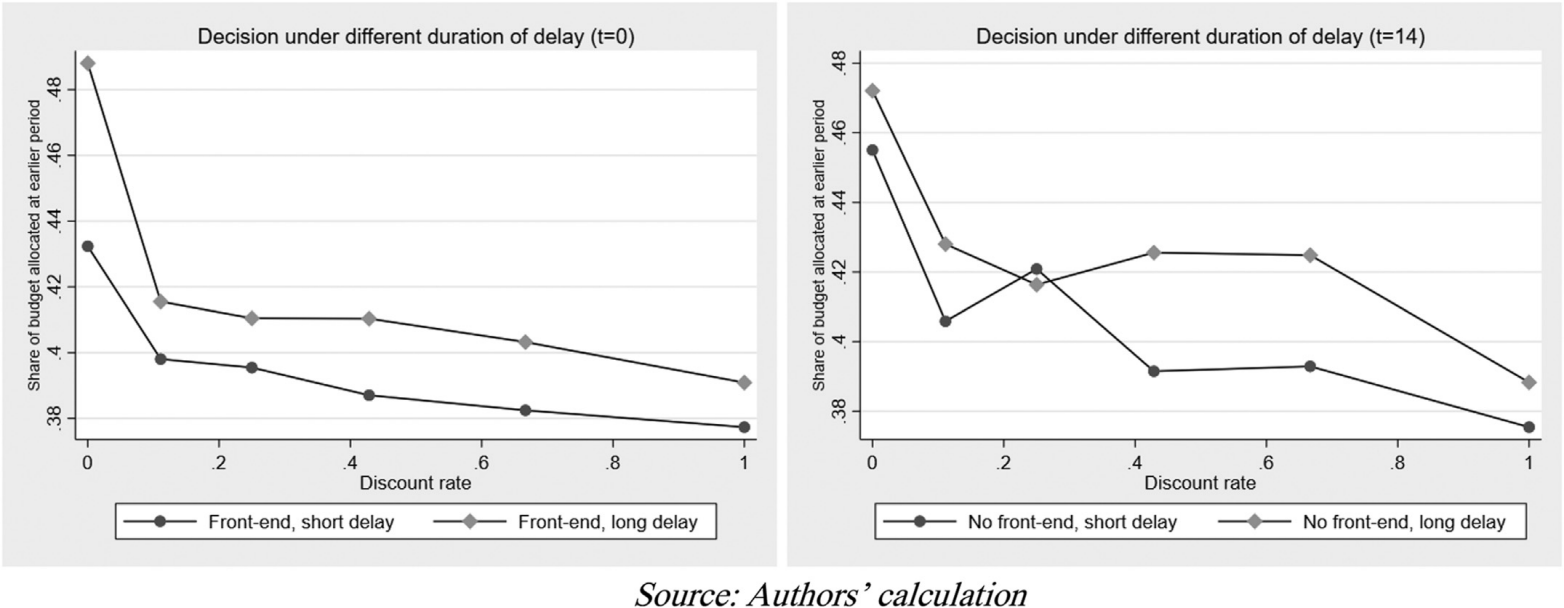
Decision-making under a different discount rate.

**Figure 6:**
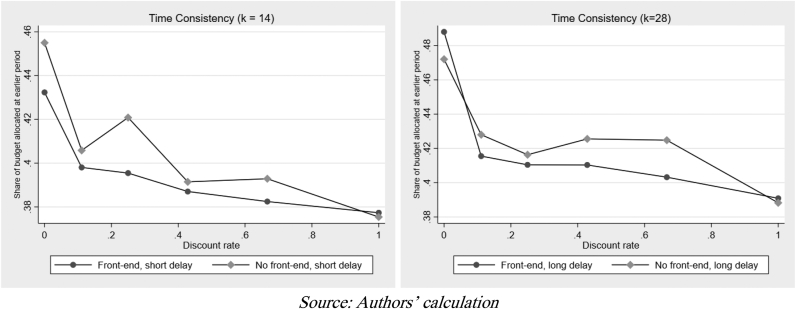
Time consistency under different delay period.

Figure 5 explains how choice is made across different delayed periods before payoffs. Theoretically, longer delays between payoffs should make students less willing to postpone payments into the future. Both graphs are consistent with theory; regardless of when the early rewards are offered, in the aggregate, students are more willing to defer their rewards when the delay is shorter. Meanwhile, Figure 6 shows whether, at the aggregate level, students’ budget allocations were smaller when rewards were paid immediately. The figure indicates future-biasedness or the existence of delayed gratification on an aggregate level. Regression analysis – through the OLS and ICR – will extract the parameter of present bias $\;\left(\hat{\beta }\right)$, along with the CRRA utility curvature $\left(\hat{\alpha }\right)\;$ and daily discount rate $\left(\hat{\delta }\right)$ for further conclusions.

Table 2 displays each parameter obtained through different regression techniques. Apart from the results from the ICR in Column (4), the CRRA utility curvature parameter is below 1, which indicates that budgets are distributed in both periods. The daily discount rate ranges from 0.982 to 0.993, which indicates an effective yearly rate of 36,657.3% to 37,016.9%. These values, however, should be inferred carefully. Although the values are incredibly high for an annual rate, it is because the choice is based on a relatively short delay (14 and 28 days). A longer delay between payments may lead to a lower annual rate, as shown in Wang et al. (2016) study of cross-country time preferences.

**Table 2:** Parameters from time elicitation.

Parameter	(1)	(2)	(3)	(4)
	OLS	ICR	OLS	ICR
$\hat{\alpha }$	0.632***	0.445	-0.314***	-2.633***
	(0.029)	(0.067)	(0.058)	(0.439)
$\hat{\delta }$	0.989***	0.983***	0.993***	0.982***
	(0.003)	(0.004)	(0.001)	(0.005)
$\hat{\beta }$	1.053***	1.082***	1.037***	1.104***
	(0.040)	(0.062)	(0.022)	(0.064)

*N*	7,389	7,389	7,389	7,389

Standard error in parentheses; *p < 0.10, **p < 0.05, ***p < 0.010.

Columns (1) and (2): Regression with restriction $\omega_{1}\;=\;\omega_{2}=0$; Column (3) and (4): Regression with restriction $\omega_{1}\;=\;\omega_{2}=\;$ average daily allowance spent.

Source: Authors' estimation.

Moreover, the focus of this study is not the annual rate but the parameter of present bias which reflects the ability to delay gratification. All regression techniques show that $\hat{\beta }$ is above 1. Consistent with the earlier analyses in Figure 5 and Figure 6, this parameter indicates that, on average, students are future-biased or have the ability to delay gratification.

### 4.2 Estimation results

Table 3 shows the regression results used to analyze the impact of mindset on learning outcome. After controlling for students’ socio-economic statuses and other factors, a greater higher mindset score or stronger beliefs in a growth mindset was associated with better math scores at a 10% significance level. Moreover, after controlling with a delayed gratification as well as socio-economic conditions of students, we also confirm that a higher growth mindset is associated with a higher math score at a 5% significance level (Table 4). We therefore argue that stronger tendencies towards a growth mindset may be important in improving students’ learning outcomes. This corroborates results of past studies which have shown that a growth mindset is positively associated with resilience, school engagement, and psychological well-being (Zeng et al., 2016). Factors such as higher resilience will, in turn, lead to better academic achievement (Aronson et al., 2002). A stronger growth mindset can also eliminate a potential mental barrier as students believe in their ability to achieve better outcomes by expanding their intelligence and capacity.

**Table 3:** Estimation results: Learning outcome and mindset.

	Math Score	
	(1)	(2)
Mindset score	0.224	0.263*
	(0.141)	(0.145)
Dummy for school 2		-6.433***
		(2.257)
Dummy for school 3		-7.246***
		(2.140)
Dummy for school 4		-4.074*
		(2.352)
Age		2.026**
		(0.830)
Male		0.573
		(1.418)
Number of family members		-0.210
		(0.295)
Index of electronic devices ownership		0.684
		(2.442)
Dummy for negative attitude toward mathematics		-3.247**
		(1.393)
Student's satisfaction on teacher's pedagogy		-2.173**
		(1.049)
Student's perspective on class condition		1.174
		(0.965)
Constant	29.500***	9.870
	(6.008)	(13.707)
*N*	309	275
*R*^2^	0.009	0.109

Standard errors in parentheses.

* = significant at 10% significance level.

** = at 5% significance level.

*** = at 1% significance level.

Source: Authors' estimation.

**Table 4:** Estimation results: Parameter of present bias (delayed gratification), mindset score and learning outcome.

	Math Score					
	(1)	(2)	(3)	(4)	(5)	(6)
Parameter of present bias ($\hat{\beta })$^a^	0.463**	4.56e-13	0.488**	-2.43e-13	0.497**	3.00e-6
	(0.222)	(0.000)	(0.212)	(0.000)	(0.222)	(0.000)
Mindset score			0.259*	0.250*	0.337**	0.313**
			(0.149)	(0.148)	(0.152)	(0.152)
Dummy for school 2					-6.911***	-6.882***
					(2.395)	(2.166)
Dummy for school 3					-7.839***	-7.786***
					(2.272)	(2.381)
Dummy for school 4					-4.788*	-4.406**
					(2.474)	(2.234)
Age					2.043**	1.994**
					(0.846)	(0.932)
Male					0.730	0.531
					(1.451)	(1.510)
Number of family members					-0.188	-0.207
					(0.303)	(0.285)
Index of electronic devices ownership					0.959	1.104
					(2.531)	(2.438)
Dummy for negative attitude toward mathematics					-3.029**	-3.277**
					(1.469)	(1.524)
Student's satisfaction on teacher's pedagogy					-2.313**	-2.298**
					(1.102)	(1.085)
Student's perspective on class condition					1.016	1.179
					(1.011)	(1.062)
Constant	38.300***	38.960***	27.480***	28.530***	6.978	8.845
	(0.818)	(0.715)	(6.432)	(6.184)	(14.196)	(15.872)
*N*	295	294	295	294	263	262
*R*^2^	0.007	0.000	0.018	0.010	0.124	0.118

Standard errors in parentheses.

* = significant at 10% significance level.

** = at 5% significance level.

*** = at 1% significance level.

Source: Authors' estimation.

a Column (1), (3), and (5): $\hat{\beta }$ is estimated using OLS; Column (2), (4), and (6): $\hat{\beta }$ is estimated using Interval Censored Regression (ICR).

Our study also suggests that students with negative attitudes towards mathematics tend to perform worse than students with neutral or positive attitudes. They score 3 points lower compared to students with non-negative attitudes. This result is similar to that found by Neale et al. (1970), who show that significant positive correlations exist between attitude towards school subjects and the test scores of those subjects. As argued by Abu-Hilal (2000), having negative attitudes would lead to lower levels of aspiration, i.e. educational goals, which in turn impacts student achievement. Several studies have also shown that aspiration or motivation can affect achievement through many channels, such as better study strategies (Sobral, 2004) and greater study effort (Kusurkar et al., 2013). As such, fostering more positive attitudes toward subjects might be crucial to improve academic performance.

Table 4 presents the regression results between parameters of present bias, that were estimated using the OLS and ICR, and learning outcome. Inclusion of the present bias variables in the model strengthens the correlation between mindset and learning outcomes. The positive value of the coefficient for $\hat{\beta }$ from both OLS and ICR models indicates that the greater the future bias (or better ability to resist temptation) a student has, the higher their math score. However, the OLS and ICR yield different results regarding the significance of delayed gratification, even after controlling for mindset. Hence, there is no strong evidence that delayed gratification is associated with better education performance. There are several reasons that may explain this. The mathematical questions asked in this study were mostly elementary-level problems, which students learned at least two years prior. This time gap may mean that the penalty from students’ inability to delay gratification no longer exists, and their understanding of past material is thus no longer influenced by their time preference. More specifically, because understanding junior high-school-level problems require an understanding of elementary-level concepts, students may have been able to catch-up and nullify the impact of their inability to delay gratification. This argument presents a research gap which should be further studied, i.e., how delayed gratification explains variation in the learning outcomes of more-recently taught materials, and how the impact of delayed gratification varies on materials taught in different years. Such variations cannot be captured in this study because the differences in learning materials between schools (in the same time period) may cause biases in the measurement of learning outcomes.

Another reason is that academic achievement may be correlated with delayed gratification, but only up to a certain level. Watts et al. (2018) show that children who waited for more than 20 s in the “Marshmallow Test” did not necessarily have better academic achievement in the future. This result implies that the ability to resist temptation is required, but how it affects academic outcome and the extent of the effect depends on other variables, such as which developmental stage students are in. Developmental stages may also explain why different aspects of human behaviors, i.e., mindset and delayed gratification, affect learning outcomes differently. Adolescent learning outcomes may be better explained by the ability to delay gratification at younger age (Mischel et al., 1988), but Atance and Jackson (2009) argued that children's future-oriented behaviors, including delaying gratification, develop substantially as they get older. As such, the existence of delayed gratification among older children may be a product of increasing age rather than actual future-oriented behavior. On the contrary, as shown in various studies, the impact of a growth mindset towards learning outcomes are more consistent across different age groups, e.g. during adolescence (Burnette et al., 2017; Claro et al., 2016) and among young adults at the university level (Broda et al., 2018).

The absence of meaningful impacts from delayed gratification towards learning outcomes can also be justified by recent theoretical interpretations of delayed gratification, such as theories that proclivity towards immediate gratification can also indicate adaptive responses instead of poor self-control or irrationality (Duran and Grissmer, 2020; Lee and Carlson, 2015). With the majority of the students in our school sample are coming from low-income families, preferences for immediate gratification may be due to the adaptation process regarding reward uncertainty, not due to failure of self-control in the learning process.

Finally, Tables 3 and 4 present some results which may be counterintuitive. Both tables show that students’ satisfaction towards their teachers’ pedagogy is negatively associated with their math scores. Similar results were found by Coe et al. (2014), who show that popular teaching practices such as using praise (Hattie and Timperley, 2007) and presenting information in a way that students preferred (Howard-Jones, 2014; Pashler et al., 2009) may be ineffective in raising learning outcomes. Hence, student satisfaction with teaching practices may not always lead to better academic performance; popular teaching practices does not equate to good practices. It is important to note, however, that this condition cannot be generalized for every type of school. Some high-performing schools may be able to implement good teaching practices that students prefer while simultaneously improving their academic performance – but such extensions are beyond the scope of our study.

## 5 Conclusion

This field survey was conducted to examine whether a strong relationship exists between growth mindsets, delayed gratification, and learning outcomes. Several insightful findings are highlighted: first, mindsets can play a part in affecting learning outcomes, though results were relatively weak. A stronger belief in the growth mindset is positively associated with a better learning outcome. Hence, going forward, it may be essential to address this issue by encouraging students to believe in themselves and to believe that intelligence can grow. Specifically, for low-performing schools, encouraging a growth mindset among students by incorporating it into curriculums or in the pedagogy may be effective in fostering greater student achievement. Second, there was no strong proof that delayed gratification explained differences in the mathematical learning outcome. Despite this, delayed gratification may still have an impacts on learning outcomes, especially for more recently studied subjects. Thus, delayed gratification should not be treated as a trivial issue, but rather must be explored further to obtain broader and stronger conclusions. This study also brings attention to a fundamental issue in the Indonesian education system. As the problem set used to gauge learning outcomes was designed around elementary school questions, the low average math score indicates that students did not really understand what they had learned in elementary school. Further studies must be conducted to identify the source of this discrepancy between curricula and learning outcomes.

Moreover, other psychological factors are important in determining a student's academic achievements. Students’ satisfaction with their teachers and their perception towards their subjects can also affect their academic achievements. Students who are satisfied with their teacher's delivery methods tend to perform poorly, indicating that teaching practices which are popular among students may not positively impact achievement. Meanwhile, when students negatively perceive a subject, e.g. believe that mathematics is complicated, they tend to have lower academic achievement. These additional findings increase the urgency to include the role of student behaviors in policy planning.

This study, however, has several limitations. First, the school selection for this study is specific to private, low-performing schools. Therefore, the impact of a growth mindset and delayed gratification cannot be assumed equal for other schools with different characteristics. To generalize the effect, a larger-scale study that includes a broader variety of schools should be conducted to improve the reliability of results and to add robustness to the weak associations found in this study. Second, the design for the mathematical test is based on elementary-level questions that require students to understand, or at least remember, what they studied at least two years prior. Therefore, future studies should focus on how student behaviors influence outcomes based on topics that they studied more recently in order to identify differences in short-term and long-term impacts. Third, our study is focuses only on one aspect of cognitive ability, i.e. mathematical competency. As such, the literature would benefit from research that explores different cognitive abilities to identify whether similar patterns exist surrounding the impact of growth mindsets and delayed gratification towards a more complete set of cognitive skills.

## Declarations

### Author contribution statement

Fandy Rahardi: Conceived and designed the experiments; Performed the experiments; Analyzed and interpreted the data; Contributed reagents, materials, analysis tools or data; Wrote the paper.

Teguh Dartanto: Conceived and designed the experiments; Analyzed and interpreted the data; Contributed reagents, materials, analysis tools or data; Wrote the paper.

### Funding statement

This work was supported by The HIBAH PUTI Q1 2020 of Universitas Indonesia: NKB-1435/UN2.RST/HKP.05.00/2020.

### Data availability statement

Data included in article/supplementary material/referenced in article.

### Declaration of interests statement

The authors declare no conflict of interest.

### Additional information

No additional information is available for this paper.

## Appendix A Supplementary data

Appendix for Heliyon -Rahardi & Dartanto.docx Appendix for Heliyon -Rahardi & Dartanto.docx

Dataset - Growth Mindset.dta Dataset - Growth Mindset.dta

## Footnotes

1 Ministry of Education and Culture of Indonesia (2020) defines national exam score into 4 categories; A (85 or above; out of 100), B (70–85), C (55–70); and less than 55 (D or failing grade).

2 The concept of present bias or lack of delayed gratification has been found in many studies, such as money-time pair experiments done by Benhabib, Bisin, and Schotter (Benhabib et al., 2010) and Balakrishnan et al. (2020).

3 Average national exam score of 4 school in 2017 is 38.32.

4 Average percentage of certified teacher at 4 schools in 2018 is 48.12%.

5 Indonesia's education system that is managed by both public and private institutions comprises four levels of education: primary (grades 1–6), junior secondary (grades 7–9), senior secondary (grades 10–12), and higher education. The private schools contribute a significant role, accounting for around 48% of all schools, 31% of all students, and 38% of all teachers (World Bank, 2010). In the case of junior secondary school, the proportion of private schools is around 41% (27,544) of total junior secondary schools (Statistics Indonesia, 2019). Rosser (2018) shows that religiously oriented schools that are mostly associated with Indonesia's two major Islamic social organizations, Muhammadiyah and Nahdlatul Ulama dominated the private educational system. Moreover, there is a great variation of quality among both public and private schools in which public schools are generally considered to be of higher quality than private schools due to better resources and more well-trained teachers (Berkhout and Tresnatri, 2020). Students who were not accepted to public schools had to attend private school because the free public junior secondary schools only serve about 70% of student nationally (Berkhout and Tresnatri, 2020).

6 As our department did not have an official review board, a panel at the Department of Economics, Universitas Indonesia reviewed the design of the research survey to ensure no violation of ethical conduct.

7 The detailed questionnaire (translated) can be found in the Appendix.

8 A choice is considered to be consistent if c_t_ is non-decreasing as implicit discount rate increase monotonically moving down the list.
