# Supplementary material for: Growth mindset, delayed gratification, and learning outcome: evidence from a field survey of least-advantaged private schools in Depok-Indonesia
Source: Heliyon. 2021 Apr 24;7(4):e06681. doi: 10.1016/j.heliyon.2021.e06681 (PMC8099761; doi:10.1016/j.heliyon.2021.e06681)
Supplement: Appendix for Heliyon -Rahardi & Dartanto.docx [file mmc1.docx]

**APPENDICES**

Rahardi, F & Dartanto, T. Growth Mindset, Delayed Gratification, and Learning Outcome: Evidence from a Field Survey of Least-Advantaged Private Schools in Depok-Indonesia

**Appendix 1. Questionnaire**

Dear parents,

Please allow me to introduce myself. I am Fandy Rahardi, final-year student from Faculty of Economics and Business at University of Indonesia. Currently, my team and I are conducting a research on the role of non-cognitive ability towards learning outcome. As such, I would like to ask for your permission to allow your child to become a participant in this study. This study will be conducted on a weekly basis, for two weeks starting from now.

This study aims to analyse how student’s behaviour might affect their academic achievement. Student will fill several questionnaires and answer a test to analyse their psychological background and basic knowledge.

We have asked for the permission from the school to do this study to minimize the negative impact towards your child learning duration at the school. All participant in this study will receive a souvenir as a token of appreciation for the allocated time. We are doing our utmost best to ensure the result of this study would not affect school’s assessment. We also hope that the outcome of this study can be used to improve student’s learning activity in the future.

If this form has been filled, please give this form back to your child to allow us to tabulate your response. For further information, please kindly contact me through the following address.

Thank you for your attention and cooperation.

I hereby declared that students with the details as follows:

Name of parents :

Phone number :

Student’s name :

Address :

By signing this document, I allowed my son/daughter to become the participant of this study without any coercion from any parties. If I and/or my child are being harmed in any process in this study, I have the rights to immediately withdraw my child’s participation in this study.

**Form A**

| No | Payment | 1^st^ option | 2^nd^ option | 3^rd^ option | 4^th^ option | 5^th^ option |
| --- | --- | --- | --- | --- | --- | --- |
| 1 | Today  **and** 2 weeks from now | 40,000  0 | 30,000  10,000 | 20,000  20,000 | 10,000  30,000 | 0  40,000 |
|  | Check only one option |  |  |  |  |  |
|  |  |  |  |  |  |  |
| 2 | Today  **and** 2 weeks from now | 36,000  0 | 27,000  10,000 | 18,000  20,000 | 9,000  30,000 | 0  40,000 |
|  | Check only one option |  |  |  |  |  |
|  |  |  |  |  |  |  |
| 3 | Today  **and** 2 weeks from now | 32,000  0 | 24,000  10,000 | 16,000  20,000 | 8,000  30,000 | 0  40,000 |
|  | Check only one option |  |  |  |  |  |
|  |  |  |  |  |  |  |
| 4 | Today  **and** 2 weeks from now | 28,000  0 | 21,000  10,000 | 14,000  20,000 | 7,000  30,000 | 0  40,000 |
|  | Check only one option |  |  |  |  |  |
|  |  |  |  |  |  |  |
| 5 | Today  **and** 2 weeks from now | 24,000  0 | 18,000  10,000 | 12,000  20,000 | 6,000  30,000 | 0  40,000 |
|  | Check only one option |  |  |  |  |  |
|  |  |  |  |  |  |  |
| 6 | Today  **and** 2 weeks from now | 20,000  0 | 15,000  10,000 | 10,000  20,000 | 5,000  30,000 | 0  40,000 |
|  | Check only one option |  |  |  |  |  |

**Form B**

| No | Payment | 1^st^ option | 2^nd^ option | 3^rd^ option | 4^th^ option | 5^th^ option |
| --- | --- | --- | --- | --- | --- | --- |
| 1 | 2 weeks from now  **and** 4 weeks from now | 40,000  0 | 30,000  10,000 | 20,000  20,000 | 10,000  30,000 | 0  40,000 |
|  | Check only one option |  |  |  |  |  |
|  |  |  |  |  |  |  |
| 2 | 2 weeks from now  **and** 4 weeks from now | 36,000  0 | 27,000  10,000 | 18,000  20,000 | 9,000  30,000 | 0  40,000 |
|  | Check only one option |  |  |  |  |  |
|  |  |  |  |  |  |  |
| 3 | 2 weeks from now  **and** 4 weeks from now | 32,000  0 | 24,000  10,000 | 16,000  20,000 | 8,000  30,000 | 0  40,000 |
|  | Check only one option |  |  |  |  |  |
|  |  |  |  |  |  |  |
| 4 | 2 weeks from now  **and** 4 weeks from now | 28,000  0 | 21,000  10,000 | 14,000  20,000 | 7,000  30,000 | 0  40,000 |
|  | Check only one option |  |  |  |  |  |
|  |  |  |  |  |  |  |
| 5 | 2 weeks from now  **and** 4 weeks from now | 24,000  0 | 18,000  10,000 | 12,000  20,000 | 6,000  30,000 | 0  40,000 |
|  | Check only one option |  |  |  |  |  |
|  |  |  |  |  |  |  |
| 6 | 2 weeks from now  **and** 4 weeks from now | 20,000  0 | 15,000  10,000 | 10,000  20,000 | 5,000  30,000 | 0  40,000 |
|  | Check only one option |  |  |  |  |  |

**Form C**

| No | Payment | 1^st^ option | 2^nd^ option | 3^rd^ option | 4^th^ option | 5^th^ option |
| --- | --- | --- | --- | --- | --- | --- |
| 1 | Today  **and** 4 weeks from now | 40,000  0 | 30,000  10,000 | 20,000  20,000 | 10,000  30,000 | 0  40,000 |
|  | Check only one option |  |  |  |  |  |
|  |  |  |  |  |  |  |
| 2 | Today  **and** 4 weeks from now | 36,000  0 | 27,000  10,000 | 18,000  20,000 | 9,000  30,000 | 0  40,000 |
|  | Check only one option |  |  |  |  |  |
|  |  |  |  |  |  |  |
| 3 | Today  **and** 4 weeks from now | 32,000  0 | 24,000  10,000 | 16,000  20,000 | 8,000  30,000 | 0  40,000 |
|  | Check only one option |  |  |  |  |  |
|  |  |  |  |  |  |  |
| 4 | Today  **and** 4 weeks from now | 28,000  0 | 21,000  10,000 | 14,000  20,000 | 7,000  30,000 | 0  40,000 |
|  | Check only one option |  |  |  |  |  |
|  |  |  |  |  |  |  |
| 5 | Today  **and** 4 weeks from now | 24,000  0 | 18,000  10,000 | 12,000  20,000 | 6,000  30,000 | 0  40,000 |
|  | Check only one option |  |  |  |  |  |
|  |  |  |  |  |  |  |
| 6 | Today  **and** 4 weeks from now | 20,000  0 | 15,000  10,000 | 10,000  20,000 | 5,000  30,000 | 0  40,000 |
|  | Check only one option |  |  |  |  |  |

**Form D**

| No | Payment | 1^st^ option | 2^nd^ option | 3^rd^ option | 4^th^ option | 5^th^ option |
| --- | --- | --- | --- | --- | --- | --- |
| 1 | 2 weeks from now  **and** 6 weeks from now | 40,000  0 | 30,000  10,000 | 20,000  20,000 | 10,000  30,000 | 0  40,000 |
|  | Check only one option |  |  |  |  |  |
|  |  |  |  |  |  |  |
| 2 | 2 weeks from now  **and** 6 weeks from now | 36,000  0 | 27,000  10,000 | 18,000  20,000 | 9,000  30,000 | 0  40,000 |
|  | Check only one option |  |  |  |  |  |
|  |  |  |  |  |  |  |
| 3 | 2 weeks from now  **and** 6 weeks from now | 32,000  0 | 24,000  10,000 | 16,000  20,000 | 8,000  30,000 | 0  40,000 |
|  | Check only one option |  |  |  |  |  |
|  |  |  |  |  |  |  |
| 4 | 2 weeks from now  **and** 6 weeks from now | 28,000  0 | 21,000  10,000 | 14,000  20,000 | 7,000  30,000 | 0  40,000 |
|  | Check only one option |  |  |  |  |  |
|  |  |  |  |  |  |  |
| 5 | 2 weeks from now  **and** 6 weeks from now | 24,000  0 | 18,000  10,000 | 12,000  20,000 | 6,000  30,000 | 0  40,000 |
|  | Check only one option |  |  |  |  |  |
|  |  |  |  |  |  |  |
| 6 | 2 weeks from now  **and** 6 weeks from now | 20,000  0 | 15,000  10,000 | 10,000  20,000 | 5,000  30,000 | 0  40,000 |
|  | Check only one option |  |  |  |  |  |

Please put a check mark on the column that indicates whether you agree or disagree with the following statement(s)

| No | Statement | Highly agree | Agree | Disagree | Highly disagree |
| --- | --- | --- | --- | --- | --- |
| 1 | Your intelligence is something very basic about you that you can’t change very much |  |  |  |  |
| 2 | No matter how much intelligence you have, you can always change it quite a bit. |  |  |  |  |
| 3 | You can always substantially change how intelligent you are. |  |  |  |  |
| 4 | You are a certain kind of person, and there is not much that can be done to really change that. |  |  |  |  |
| 5 | You can always change basic things about the kind of person you are. |  |  |  |  |
| 6 | Music talent can be learned by anyone. |  |  |  |  |
| 7 | Only a few people will be truly good at sports – you have to be “born with it.” |  |  |  |  |
| 8 | Math is much easier to learn if you are male or maybe come from a culture who values math |  |  |  |  |
| 9 | The harder you work at something, the better you will be at it. |  |  |  |  |
| 10 | No matter what kind of person you are, you can always change substantially. |  |  |  |  |
| 11 | Trying new things is stressful for me and I avoid it. |  |  |  |  |
| 12 | Some people are good and kind, and some are not – it’s not often that people change. |  |  |  |  |
| 13 | I appreciate when people, parents, coaches, teachers give me feedback about my performance. |  |  |  |  |
| 14 | I often get angry when I get feed back about my performance. |  |  |  |  |
| 15 | All human beings without a brain injury or birth defect are capable of the same amount of learning. |  |  |  |  |
| 16 | You can learn new things, but you can’t really change how intelligent you are |  |  |  |  |
| 17 | You can do things differently, but the important parts of who you are can’t really be changed |  |  |  |  |
| 18 | Human beings are basically good, but sometimes make terrible decisions. |  |  |  |  |
| 19 | An important reason why I do my school work is that I like to learn new things. |  |  |  |  |
| 20 | Truly smart people do not need to try hard. |  |  |  |  |

**Research Questionnaire**

Thank you for your willingness to participate in this study. We guarantee the confidentiality of any information you filled in this questionnaire, and it will only be used solely for the purpose of this research and not to harm participant in any forms.

**Section I: Personal Identity**

1. Full name :
2. Gender : Male/Female
3. Date, Place of Birth:
4. Religion : Islam / Christian / Catholic / Hindu / Buddha / Others……………
5. Ethnicity :
6. Parent’s age
   1. Father : …………… years old
   2. Mother : …………… years old
7. Parent’s education
   1. Father:

(1) Not graduated from elementary school / (2) Elementary school or similar / (3) Junior high school or similar / (4) Senior high school or similar / (5) Diploma / (6) Bachelor / (7) Master’s degree / (8) Doctoral degree / (9) Others ………

- 1. Mother:

(1) Not graduated from elementary school / (2) Elementary school or similar / (3) Junior high school or similar / (4) Senior high school or similar / (5) Diploma / (6) Bachelor / (7) Master’s degree / (8) Doctoral degree / (9) Others ………

1. Parent’s occupation
   1. Father:

(1) Unemployed / (2) Civil servant / (3) Private employees / (4) Self-employed / (9) Others ………

- 1. Mother:

(1) Unemployed / (2) Civil servant / (3) Private employees / (4) Self-employed / (9) Others ………

1. Is your biological parents still alive?
2. Yes / (2) Only father / (3) Only mother / (4) None
3. Status of parents
   1. Father :

(1) Biological father / (2) Foster-father / (3) Stepfather / (9) Others…

- 1. Mother :
     1. Biological mother / (2) Foster-mother / (3) Stepmother / (9) Others…

1. Family member:
   1. Number of male siblings:
   2. Number of female siblings:
2. With whom did you stay at home? (Can check more than one)

(1) Parents / (2) Siblings / (3) Stepsiblings / (4) Grandparents / (9) Others……

1. How many members of **extended family** are staying in your home?

(*Extended family consists of parents, siblings, grandparents, uncle, aunt, or cousin)*

**Section II: Facilities & Infrastructure**

1. Do you have the following item(s) at home?
   1. Main textbook: (1) Yes / (2) No
   2. Reference book: (1) Yes / (2) No
   3. Handphone: (1) None / (2) Owned by parents / (3) Owned by siblings / (4) Privately owned
   4. Computer/laptop: (1) None / (2) Owned by parents / (3) Owned by siblings / (4) Privately owned
2. Do you use gadget to look for information and study materials?
   1. Handphone: (1) Yes / (2) No
   2. Laptop: (1) Yes / (2) No
3. Do you study independently other at school? (1) Yes / (2) No
   1. If yes, how long on average did you study in each day?
4. Do you take tutoring session? (1) Yes / (2) No
5. Do you have a private teacher to study at home? (1) Yes / (2) No
6. Do you earn pocket money from parents? (1) Yes / (2) No
   1. If yes, how is the mechanism?
      1. Daily / (2) Weekly / (3) Monthly / (9) Others ……
7. On average, how much of your pocket money you spent in a day? Rp ………… - Rp …………
8. Did you save part of your pocket money? (1) Yes / (2) No
   1. If yes, how much money did you spent (on average) in a day?
9. How did you go to school? (1) Walking / (2) Public transportation / (3) Shuttle / (4) Private vehicle / (9) Others ……
10. How much money did you spent for transportation in a day? (if any)
11. How much money did you or your parent spent for your school monthly fee?
12. Do you receive any external funding for the school fee? (Can check more than one)

(1) *Bantuan Siswa Miskin* / (2) *Kartu Indonesia Pintar* / (3) Scholarship ……………… /

(9) Others………

1. How much is your parent’s income in a month?
2. Did you work part-time outside of the school duration?

**Section III: Personal**

1. What is your goals?
2. Do you have a role model? 1) Yes / (2) No
   1. If yes, who is it?
      1. Parents / (2) Relatives / (3) Celebrities / (4) Athlete / (5) Government officials / (6) Artist / (9) Others ……
3. What subject you **like** the most?
4. Math / (2) Natural Science / (3) Social Science / (4) Language / (5) Arts / (6) Sports / (9) Others ……
5. What subject you **hate** the most?
6. Math / (2) Natural Science / (3) Social Science / (4) Language / (5) Arts / (6) Sports / (9) Others ……
7. In what years did you graduate from elementary school?
8. Have you ever retained in the same education level?
9. In what years did you enter junior high school?
10. What is your mathematics national exam score at elementary level? (If you forget the result, please choose the range that you think is the closest one)
11. Less than 3 / (2) 3 to 3,99 / (3) 4 to 4,99 / (4) 5 to 5,99 / (5) 6 to 6,99 / (6) 7 to 7,99 / (7) 8 to 8,99 / (8) Equal or more than 9
12. Who choose the school you are currently enrolled in?
13. Myself / (2) Parent’s decision / (3) After discussion with parents / (9) Others …
14. From scale of 1 to 5, how do you think is the relationship **between you and your parents**?

| Very bad | Bad | Average | Good | Very good |
| --- | --- | --- | --- | --- |
| 1 | 2 | 3 | 4 | 5 |

1. From scale of 1 to 5, how do you think is the relationship **between your parents**?

| Very bad | Bad | Average | Good | Very good |
| --- | --- | --- | --- | --- |
| 1 | 2 | 3 | 4 | 5 |

**Section IV: Perspectives on Educational Issue**

1. From scale of 1 to 5, **how well did you understand** content of mathematics being taught at school?

| Very bad | Bad | Neutral | Good | Very good |
| --- | --- | --- | --- | --- |
| 1 | 2 | 3 | 4 | 5 |

1. From scale of 1 to 5, **how difficult** do you think the content of mathematics being taught at school?

| Very hard | Hard | Average | Easy | Very easy |
| --- | --- | --- | --- | --- |
| 1 | 2 | 3 | 4 | 5 |

1. From scale of 1 to 5, **how is the quality of your classroom**?

| Very bad | Bad | Average | Good | Very good |
| --- | --- | --- | --- | --- |
| 1 | 2 | 3 | 4 | 5 |

1. From scale of 1 to 5, **how is the quality of your school facilities**?

| Very hard | Hard | Average | Easy | Very easy |
| --- | --- | --- | --- | --- |
| 1 | 2 | 3 | 4 | 5 |

1. From scale of 1 to 5, **how satisfied are you with the teaching method at school**?

| Very dissatisfied | Dissatisfied | Neutral | Satisfied | Very satisfied |
| --- | --- | --- | --- | --- |
| 1 | 2 | 3 | 4 | 5 |

1. From scale of 1 to 5, **how important is the quality of classroom?**

| Extremely not important | Not important | Neutral | Important | Extremely important |
| --- | --- | --- | --- | --- |
| 1 | 2 | 3 | 4 | 5 |

1. From scale of 1 to 5, **how important is the quality of school facilities?**

| Extremely not important | Not important | Neutral | Important | Extremely important |
| --- | --- | --- | --- | --- |
| 1 | 2 | 3 | 4 | 5 |

1. From scale of 1 to 5, **how important is the quality of teaching method?**

| Extremely not important | Not important | Neutral | Important | Extremely important |
| --- | --- | --- | --- | --- |
| 1 | 2 | 3 | 4 | 5 |

1. From scale of 1 to 5, **how much is your parent’s attention toward your education?**

| Extremely not concerned | Not concerned | Neutral | Concerned | Extremely concerned |
| --- | --- | --- | --- | --- |
| 1 | 2 | 3 | 4 | 5 |

Thank you for filling this questionnaire. If all questions have been answered, please give this questionnaire back to the surveyor team in order to trade it with the souvenir

## Appendix 2. Notes on Interval Censored Regression

In the Equation 3, what we want to measure is ${y_{i}^{*}=ln\left( \frac{C_{t}-\omega_{1}}{C_{t+k}-\omega_{2}} \right)}_{i}$, where

$${ln\left( \frac{C_{t}-\omega_{1}}{C_{t+k}-\omega_{2}} \right)}_{i}=\gamma_{1}{1_{t=0}}_{i}+\gamma_{2}k_{i}+\gamma_{3}{ln(1+r)}_{i}$$

Where $\gamma_{1}=\frac{(ln\beta)}{\alpha-1}, \gamma_{2}=\frac{ln\delta}{\alpha-1}, \mathrm{and} \gamma_{3}=\left( \frac{1}{\alpha-1} \right)$. However, as individuals are constrained with a limited amount of options, exactly five options for each decision, from the five choices the value of y* can be determined as following:

$$y^{*}\left\{ \begin{aligned} 1 if y^{*}>I_{1} \\ 2 if I_{1}>y^{*}>I_{2} \\ 3 if I_{2}>y^{*}>I_{3} \\ 4 if I_{3}>y^{*}>I_{4} \\ 5 if I_{4}>y^{*} \end{aligned} \right.$$

where,

$$l_{i,1}=P\left( c_{1}=1 \right)=P\left( y^{*}>I_{1} \right)=P(\gamma_{1}{1_{t=0}}_{i}+\gamma_{2}k_{i}+\gamma_{3}{\ln\left( 1+r \right)}_{i}>I_{1})$$

$$l_{i,2}=P\left( c_{2}=1 \right)=P\left( I_{1}>y^{*}>I_{2} \right)=P(I_{1}>\gamma_{1}{1_{t=0}}_{i}+\gamma_{2}k_{i}+\gamma_{3}{\ln\left( 1+r \right)}_{i}>I_{2})$$

$$l_{i,3}=P\left( c_{3}=1 \right)=P\left( I_{2}>y^{*}>I_{3} \right)=P\left( I_{2}>\gamma_{1}{1_{t=0}}_{i}+\gamma_{2}k_{i}+\gamma_{3}{\ln\left( 1+r \right)}_{i}>I_{3} \right)$$

$$l_{i,4}=P\left( c_{4}=1 \right)=P(I_{3}>y^{*}>I_{4})=P({I_{3}>\gamma}_{1}{1_{t=0}}_{i}+\gamma_{2}k_{i}+\gamma_{3}{\ln\left( 1+r \right)}_{i}>I_{4})$$

$$l_{i,5}=P\left( c_{5}=1 \right)=P\left( I_{4}>y^{*} \right)=P(I_{4}>\gamma_{1}{1_{t=0}}_{i}+\gamma_{2}k_{i}+\gamma_{3}{\ln\left( 1+r \right)}_{i})$$

which also can be rewritten as the following:

$$l_{i,1}=1-Ф\left( \frac{I_{1}-\left( \gamma_{1}{1_{t=0}}_{i}+\gamma_{2}k_{i}+\gamma_{3}{\ln\left( 1+r \right)}_{i} \right)}{\sigma} \right)$$

$$l_{i,2}=Ф\left( \frac{I_{1}-\left( \gamma_{1}{1_{t=0}}_{i}+\gamma_{2}k_{i}+\gamma_{3}{\ln\left( 1+r \right)}_{i} \right)}{\sigma} \right)-Ф\left( \frac{I_{2}-\left( \gamma_{1}{1_{t=0}}_{i}+\gamma_{2}k_{i}+\gamma_{3}{\ln\left( 1+r \right)}_{i} \right)}{\sigma} \right)$$

$$l_{i,3}=Ф\left( \frac{I_{2}-\left( \gamma_{1}{1_{t=0}}_{i}+\gamma_{2}k_{i}+\gamma_{3}{\ln\left( 1+r \right)}_{i} \right)}{\sigma} \right)-Ф\left( \frac{I_{3}-\left( \gamma_{1}{1_{t=0}}_{i}+\gamma_{2}k_{i}+\gamma_{3}{\ln\left( 1+r \right)}_{i} \right)}{\sigma} \right)$$

$$l_{i,4}=Ф\left( \frac{I_{3}-\left( \gamma_{1}{1_{t=0}}_{i}+\gamma_{2}k_{i}+\gamma_{3}{\ln\left( 1+r \right)}_{i} \right)}{\sigma} \right)-Ф\left( \frac{I_{4}-\left( \gamma_{1}{1_{t=0}}_{i}+\gamma_{2}k_{i}+\gamma_{3}{\ln\left( 1+r \right)}_{i} \right)}{\sigma} \right)$$

$$l_{i,5}=Ф\left( \frac{I_{4}-\left( \gamma_{1}{1_{t=0}}_{i}+\gamma_{2}k_{i}+\gamma_{3}{\ln\left( 1+r \right)}_{i} \right)}{\sigma} \right)$$

We can then obtain $\alpha=\frac{\sigma}{\gamma_{3}}+1, \beta=exp(\frac{\gamma_{1}}{\gamma_{3}}), \delta=exp(\frac{\gamma_{2}}{\gamma_{3}})$
